# Use of Dermoscopy among Greek Dermatologists in Everyday Clinical Practice: A National Questionnaire-Based Study

**DOI:** 10.3390/jcm13040972

**Published:** 2024-02-08

**Authors:** Dimitrios Sgouros, Eleni Routsi, Athina Evangelodimou, Aimilios Lallas, Zoe Apalla, Dimitrios K. Arvanitis, Georgia Pappa, Elizabeth Lazaridou, Christina Fotiadou, Georgios Evangelou, Georgios Chaidemenos, Dimitrios Ioannides, Ioannis Barkis, Konstantinos Liopyris, Electra Nicolaidou, Sofia Theotokoglou, Anna Syrmali, Alexander Stratigos, Dimitrios Rigopoulos, Alexander Katoulis

**Affiliations:** 1 2nd Department of Dermatology and Venereology, “Attikon” General University Hospital, Medical School, National and Kapodistrian University of Athens, 12462 Athens, Greece; arvandi18@yahoo.com (D.K.A.); gpappa100@gmail.com (G.P.); theotokoglousofia@gmail.com (S.T.); annasyrmali@gmail.com (A.S.); alexanderkatoulis@yahoo.co.uk (A.K.); 2 1st Department of Dermatology and Venereology, “Andreas Sygros” Hospital, Medical School, National and Kapodistrian University of Athens, 16121 Athens, Greece; routsie@gmail.com (E.R.); konstantinosliopyris@gmail.com (K.L.); electra.nicol@gmail.com (E.N.); alstrat2@gmail.com (A.S.); dimitrisrigopoulos54@gmail.com (D.R.); 3 Dermatology Department, General Hospital of Athens, Evangelismos, 11635 Athens, Greece; evangelodimou.athina@gmail.com; 4 First Dermatology Department, School of Medicine, Faculty of Health Sciences, Aristotle University of Thessaloniki, 54124 Thessaloniki, Greece; emlallas@gmail.com (A.L.); dem@auth.gr (D.I.); 5 Second Dermatology Department, School of Medicine, Faculty of Health Sciences, Aristotle University of Thessaloniki, 54124 Thessaloniki, Greece; zoimd@yahoo.gr (Z.A.); bethlaz@auth.gr (E.L.); christinafotiadou@yahoo.com (C.F.); 6 Department of Dermatology, University General Hospital of Heraklion, 71500 Heraklion, Greece; gevag600@yahoo.co.uk; 7 Private Practice, 54643 Thessaloniki, Greece; cgiorgos@otenet.gr; 8 Private Practice, 14121 Athens, Greece; barkisioannis@gmail.com

**Keywords:** dermoscopy, dermatoscopy, dermatology, skin cancer, diagnosis, dermatologist

## Abstract

**Background**: Dermatoscopy has been established as an important diagnostic tool for a wide range of skin diseases. This study aims to evaluate the use of dermatoscopy in clinical practice among Greek dermatologists. **Methods**: A nationwide questionnaire-based survey was conducted collecting data on the frequency of dermatoscopic examinations, the types of lesions examined, training and educational resources, as well as factors influencing the choice to incorporate dermatoscopy into daily clinical routines. **Results**: A total of 366 Greek dermatologists participated in the survey. Most of the respondents reported the daily use of dermatoscopy in their practice. Pigmented and non-pigmented lesions, inflammatory diseases, cutaneous infectious, hair disorders, and nail lesions were the most common indications for dermatoscopy. Factors influencing the utilization of dermatoscopy included increased diagnostic accuracy, enhanced patient care, better patient communication and general compliance, and improved satisfaction among dermatologists. **Conclusions**: This national questionnaire-based study demonstrates that dermatoscopy has become an integral part of daily dermatological practice in Greece. The findings highlight the significance of structured training and education to promote dermoscopy’s effective and routine use. Incorporating dermatoscopy into clinical practice not only improves diagnostic precision but also enhances patient care, contributing to the overall quality of dermatological services in Greece.

## 1. Introduction

Dermoscopy—also known as dermatoscopy, surface microscopy, and epiluminescence microscopy—is a non-invasive in vivo technique for the evaluation of skin lesions that allows for the visualization of microstructures of the epidermis, dermal–epidermal junction, and papillary dermis [1,2]. Dermatoscopy results in an earlier recognition of melanoma and other types of skin cancer, while reducing the number of unnecessary invasive procedures needed for diagnosis. Beyond dermatoscopy, other novel non-invasive imaging techniques such as Reflectance Confocal Microscopy and Optical Coherence Tomography serve also as additional diagnostic tools for the early detection of cutaneous malignancies [3,4]. Although histological examination and clinicopathological correlation remain the gold standard for diagnosis, dermatoscopy often enhances the earlier detection of suspicious lesions, also preventing unnecessary skin biopsies or surgical excisions of benign lesions [1,2,5].

Dermatoscopy has been shown to significantly improve the diagnosis of melanocytic lesions in clinical practice [5]. Over the past several years, the use of dermatoscopy has expanded to include utilization for the diagnosis of dermatological disorders such as inflammatory dermatoses (e.g., psoriasis and lichen planus), pigmentary disorders, infectious dermatoses, appendageal tumors, and disorders of the hair, scalp, and nails [5,6]. However, melanoma detection remains the most important clinical indication of dermatoscopy [2,3,4,6,7]. Cutaneous malignant melanoma accounts for 90% of all skin cancer mortality. The incidence has been rapidly increasing in most developed countries during the past decades [2,3,5,8]. Several methods have been used so far for physicians’ training in dermoscopy for the precise differential diagnosis of lesions and above all for the earliest possible detection of melanoma: the elementary “ABCD algorithm”, the somehow dermatoscopic equivalent of the classical ABCD clinical rule for melanoma suspicious lesions; the “ugly duckling sign” representing the comparative approach of the global dermatoscopic pattern of the patients’ lesions; the “blink diagnosis” denoting the cognitive perception where the clinician is capable of recognizing at first sight the correct diagnosis after having been exposed to the same clinical entity many times in the past; the classical “pattern analysis”, an algorithmic diagnostic procedure based on the analysis of pre-defined dermatoscopic criteria [9].

Depending on the country, dermatoscopy is used not only by dermatologists, but also by other specialists, such as general practitioners/family physicians, plastic surgeons, and pediatricians. Although dermatoscopy is used worldwide, insufficient data are available concerning the trends and limitations in the use of dermatoscopy as a diagnostic tool in everyday clinical practice among Greek dermatologists [5,6,7]. We report herein the first study evaluating the use of dermatoscopy by dermatologists in Greece.

The aim of this survey was to explore the use of dermatoscopy among Greek dermatologists and its benefits in daily practice. Furthermore, we tried to figure out the demographic and training-related factors that influence Greek dermatologists’ perceptions of the use of dermatoscopy and the potential limitations of its use.

## 2. Methods

This cross-sectional study on Greek dermatologists was conducted under the auspices of the Hellenic Society of Dermatology and Venereology (HSDV) and the Hellenic Dermoscopy Society (HDS). Responses were collected through the Google Form tool for online surveys.

The questionnaire was available online from March 2021 to December 2021 (HSDV ethics committee approval code 270/15 March 2021) consisting of 24 items regarding demographic and practice characteristics (i.e., age, gender, and prefecture of work), dermatoscopy training, and use in general.

Responders were asked to complete the survey only once for research and statistical integrity. The survey contained no identifying information to ensure anonymity. The percentages were reported based on the number of participants that had answered each question, e.g., for the prefecture of work, 366 participants responded. For this question, the percentages were extracted by dividing the number of observations by 366 (192/366 = 0.52). The responders were divided into two groups based on the answer of the first question, “Do you use dermatoscope?”. Only responders answering “YES” to this question had the opportunity to proceed to questions regarding the practice of dermatoscopy use. If the answer was negative, the responders answered an additional question regarding the reasons for why they did not use dermatoscopy and did not proceed with the rest of the questionnaire. Practice locations were categorized by prefectures, and all answers to the questions were rated on a 5-point Likert scale.

## 3. Statistical Analysis

Continuous data were given as means and standard deviations. For categorical variables, the frequencies and percentages were calculated. Fisher’s exact test and the Chi-square (X^2^) test were used to compare categorical variables. A *p*-value < 0.05 was considered statistically significant. All statistical analyses were performed using Stata/IC v. 15.1.

## 4. Results

The questionnaire was sent via email to 380 Greek dermatologists and 366 responses were received. The respondents’ demographics and answers to questions are reported in Table 1. Collectively, the majority were females (n = 238, 65%) followed by males (n = 128, 35%). Concerning the age, 60.7% of the respondents (n = 222) were between 30 and 50 years old, 35.8% (n = 131) were over 50 years old, and 3.5% (n = 13) were under 30 years old (Table 1). Most participants were working in Attiki (Athens region) (n = 193, 52.7%), followed by Thessaloniki (n = 57, 15.6) as shown in Table 1.

The type of medical facility at which respondents mainly worked was a private practice (n = 233, 63.7%), followed by a general hospital (n = 97, 26.5%), and a combination of both a private practice and general hospital (n = 23, 6.3%) (Table 1). The use of a dermatoscope in dermatological practice was reported by 93.2% of the respondents (n = 341). A small number of participants (n = 25, 6.3%) did not own a dermatoscope, although stating their intention to obtain one in the future. The reasons declared for not owning or using a dermatoscope were financial distress (n = 9, 37.5%), lack of experience (n = 6, 25%), lack of training, and (n = 2, 8.2%) lack of update, training, and experience (n = 1, 4.2%), all presented in Table 2. Of those who applied dermatoscopy in their daily routine (n = 341), the majority were women (n = 220/341, 64.5%). The most common age group of the participants was between 30 and 50 years of age (n = 209, 61.3%). Regarding the setting in which physicians worked, the largest number of respondents worked in the private sector (n = 226, 66.3%) followed by the public sector (n = 81, 23.8%). Concerning the type of dermatoscope, most of them answered that they mainly used a handheld one with polarized and nonpolarized light (n = 200, 59.2%), followed by a handheld one with nonpolarized light (n = 35, 10.4%), and digital dermoscopy (n = 19, 5.6%).

The main source of training was national conferences in Greece (74.5%), followed by residency training (60%) and dermatology books (52.1%). Most of them were satisfied (four on the five-point Likert scale) with their training (n = 154, 45.5%) (Table 3).

Most of the respondents were most familiar with using the “blink diagnosis” (71.1%), ABCD algorithm (60%), dermatoscopic “ugly duckling sign” (43.2%), and pattern analysis (10.4%) as diagnostic methods for the analysis of lesions, as shown in Table 3.

Most of the dermatologists used dermatoscopy on more than 80% of their patients examined in total (n = 133, 39.2%) and perceived that dermatoscopy reduced the average diagnosis time (n = 261, 77.7%). Table 4 presents the indications of dermatoscopy in everyday clinical practice. Specifically, all the responders used dermatoscopy for dermatologic oncology (100%), nail and hair disorders (89.1%), inflammatory diseases, and (70.4%) cutaneous infectious conditions like Molluscum Contagiosum (56.5%). After diagnosis, most of the respondents believed that dermatoscopy helped to avoid unnecessary surgical procedures (n = 219, 64.6%), to determine the therapeutic approach (n = 186, 54.9%), and to choose an accurate biopsy sample enhancing the collaboration at the level of diagnosis with the pathologist (n = 299, 89%). The vast majority of the responders (n = 327, 96.8%) also used dermatoscopy in cases other than those with a high risk for melanoma (Table 4).

Moreover, our findings revealed that almost all the participants in the study (n = 325, 96.4%) used dermatoscopy as an essential part of the patients’ follow up.

Respondents mainly believed that dermatoscopy and other related imaging methods could have been of help for patient–physician communication and patients’ general compliance (n = 319, 94.4%). A total of 61.4% of responders (n = 208) stated that they used dermatoscopy as a part of telecommunication/teleconferencing with colleagues to share diagnostic approaches. However, more than one-third of the participants did not save the dermatoscopic images (n = 123, 36.4%) despite the majority (n = 314, 93.2%) stating that recording digital images as part of a dermatoscopic medical history would have been of help in efficient patient follow-ups (Table 4). To the question of whether the dermatoscopy should be separately charged, most of the respondents answered that this should be part of the dermatological examination (n = 237, 70.5%). Concerning the training of other specialists (general practitioners, plastic surgeons, pediatricians, family physicians, etc.) and if this dermatoscopical education could contribute to the earliest diagnosis of melanoma, 53.7% (n = 181) of the respondents answered positively and 46.3% (n = 156) negatively (Table 4).

## 5. Discussion

This is the first survey in Greece evaluating the use of dermatoscopy, its benefits in daily practice, and the limiting factors for its use among Greek dermatologists. Dermatoscopy is becoming an increasingly helpful tool in the earlier recognition of different dermatological diseases such as melanoma, non-melanoma skin cancers, inflammatory dermatoses, cutaneous infections, and nail and hair disorders [6,7]. This study shed some light on the status of dermatoscopy use in Greece and underlined the necessity of better training in its use. This survey may also be utilized as a valuable tool for future strategic planning towards the optimization of dermatoscopy training and daily practice use in Greece.

Many surveys from the US, Australia, UK, Brazil, France, Canada, Taiwan, India, and Saudi Arabia have studied the use of dermatoscopy among dermatologists [10,11,12,13,14,15,16,17,18,19,20,21], as well as among GPs [22,23,24]. Our survey verified that most of the responding dermatologists (93.2%) utilized dermatoscopy in the management of their patients. Similar studies in France, Brazil, US, UK, and Saudi Arabia have shown that dermatologists apply dermatoscopy in their daily routine at a rate of 94.6%, 98%, 48%, 98,5%, and 56.9%, respectively [13,15,16,20,23]. Compared to other countries, in Greece, a high proportion of dermatologists were familiar with dermatoscopy. Venugopal et al. reported that 98% of dermatologists in Australia used a dermatoscope [11]. A high prevalence of dermatoscopy use in Australia has been confirmed by previous studies [8,9]. Australia has the highest incidence of skin cancer of any country in the world [11]. A study from Australia by Piliouras et al. found that that all trainees (100%) used dermatoscopy. Although all the trainees applied dermatoscopy in their daily routine, surprisingly, over half of them declared that they did not own a dermatoscope due to its cost. However, it seems encouraging that most institutions supply dermatoscopes for use by their doctors [18]. Based on our results, in Greece, the most important reasons for not using dermatoscopy were the financial cost (37.5%) and the lack of education and training (25%). This rate was higher than that reported in the United States (6.6%) [14]. In a similar survey in Taiwan, one-third of those who reported not using a dermatoscope declared that this was related to the cost [13]. This could be because training institutions in Greece do not supply dermatoscopes to clinicians.

The demographics of our respondents were comparable to those in similar surveys, with most of them being composed of women. This could be explained by the higher proportion of female dermatologists worldwide. Consistent with the findings from other countries, the age of our responders ranged between 30 and 50 years old. Most of the responders were working in the Athens metropolitan region (52.7%), followed by Thessaloniki (15.6%), which are the two largest cities in the country. The practice setting was mainly a private practice (63.7%) followed by a general hospital (26.5%). Similarly, according to Forsea et al. (2016), a pan-European survey covering 42 countries showed that dermatologists working in individual practices were the most likely to apply dermatoscopy [7]. The type of dermatoscope participants mostly used was a handheld one with polarized and nonpolarized light (59.2%). This could be explained due to the portability and hybridity of this device. Similarly, Barcaui et al. showed that 83% of the participants used polarized light [25].

The most frequently reported training methods included conferences (74.5%), followed by residency programs (60%) and books (52.1%). These results underlined the need for integration in dermatoscopy training, in a more intense manner, into residency programs. It is also important to highlight the importance of Greek conferences in the training of dermatoscopy. This was generally in agreement with the results of the study by Forsea et al. [7]. In most studies, conferences, books, atlases, and residency programs comprise the main reported sources of education [7,13,19,25].

Our study revealed that most of the dermatologists used dermatoscopy on more than 80% of their visiting patients. This also indicated a high degree of engagement of dermatoscopy by Greek dermatologists. Although dermatoscopy was first utilized for diagnosing pigmented lesions, in this study, most of the dermatologists used dermatoscopes for pigmented and non-pigmented lesions, inflammatory diseases, cutaneous infections, hair disorders, and nail lesions. In most other studies, clinicians used dermatoscopy for pigmented and non-pigmented lesions [13,14,25], less than for other indications. As expected, due to the low incidence of skin cancers in skins of color, Kaliyadan et al. reported that the use of dermatoscopy in India was mainly in the context of inflammatory and hair disorders, rather than tumors [26]. Except for the universal use of dermatoscopy in dermato-oncology (100%), we observed a high examination rate in appendage abnormalities (hair–nail disorders) and inflammatory dermatoses. The application of dermatoscopy beyond skin tumors is continually increasing, and our results confirmed this current trend [27].

Instead of classic algorithms like the “blink diagnosis” (71.2%), ABCD algorithm (59.8%), and ugly duckling sign (43.2%), a significantly low percentage of pattern analysis was recorded (10.5%). Simplified algorithms such as the ABCD rule, “blink diagnosis”, and ugly duckling sign were developed to facilitate the use of dermatoscopy in daily practice, especially for non-experts. However, pattern analysis remains the most reliable technique for expert dermatologists [9,27,28]. This finding was not in accordance with the current literature concerning the diagnostic algorithms used in other countries. Analytically, according to Noor O et al. and Barcaui et al., 89.5% and 63% of their participants used pattern analysis, respectively [21,25]. Based on this, a better training of Greek dermatologists in pattern analysis should be considered.

Our study also demonstrated that dermatoscopy could have a significant contribution in more efficient communication between dermatologists and patients as well as with colleagues. However, only a few of the participants declared that they saved digital dermatoscopic images as a standard procedure after patient examination. The improvement of image storage and history recording would facilitate the daily work of dermatologists, allowing for more coordinated and efficient multidisciplinary care. This could help dermatologists more effectively diagnose patients, reduce medical mistakes, and provide safer care. Based on our knowledge, this is something that has not yet been thoroughly investigated, especially from the clinicians’ perspective. Under certain conditions, Greek dermatologists believed that the basic training in dermatoscopy for other specialists (e.g., plastic surgeons, etc.) could potentially improve early melanoma diagnosis and overall skin cancer prevention. In accordance with Chappuis et al., Greek dermatologists also claimed that dermatoscopy should not be charged as an extra to the payment of the basic clinical examination [24].

This study had some potential limitations. The aforementioned factors may have caused a sampling bias between users and non-users participating in the survey, which might have led to an overestimation of dermatoscopy use. It was more likely for dermatoscopy users to respond to this survey than non-users. This survey was exclusively sent to Greek dermatologists; thus, our findings should not be generalized outside of Greece. Many studies worldwide have already provided information about dermatoscopy use among general practitioners [22,23,24]. This could also be considered for future research. Participants from the rest of Greece were fewer than those from Athens and Thessaloniki, reflecting that most training courses are commonly based in urban areas.

Despite these limitations, the study provided much information about the use of dermatoscopy among Greek dermatologists. Similar studies should be constantly conducted towards additional information and progress in the field of training and familiarization with this method.

In conclusion, this is the first published survey on the use of dermatoscopy in Greece. This study showed that dermatoscopy utilization is increasing in Greece. Considering the evidence-based advantages of dermatoscopy towards the earlier detection of skin cancers, this study could raise the awareness about the long-lasting benefits of dermatoscopy for improving dermatological care and skin cancer prevention in Greece.

## Figures and Tables

**Table 1:** Demographics (gender, age, working area, and medical facility).

**Gender (n = 366)**	**N (%)**	***p*-Value**
Male	128 (35)	
Female	238 (65)	
		0.52
**Age (Years) (n = 366)**	**N (%)**	
<30 years	13 (3.5)	
30–50 years	222 (60.7)	
>50 years	131 (35.8)	
		<0.001
**Working area (n = 366)**	**N (%)**	
Attiki (Athens)	193 (52.7)	
Thessaloniki	57 (15.6)	
Other	116 (31.7)	
**Medical facility (n = 366)**	**N (%)**	
Private practice	233 (63.7)	
General hospital	97 (26.5)	
Primary healthcare	8 (2.2)	
Both private practice and general hospital	23 (6.3)	
Other	5 (1.3)	
		<0.001

**Table 2:** Reasons for not owning/using type of dermatoscope.

**Reasons for not Owning (n = 24)**	**N (%)**
Financial reasons	9 (37.5)
Lack of experience	6 (25)
Lack of update and training	2 (8.2)
Financial reasons, lack of experience	2 (8.3)
Lack of update, training, and experience	1 (4.2)
Wrong timing	1 (4.2)
Other subspecialty	1 (4.2)
Different work field	1 (4.2)
Financial reasons, lack of experience and training	1 (4.2)
**Type of dermatoscope (n = 338)**	**N (%)**
Handheld with polarized and nonpolarized light	200 (59.2)
Handheld with nonpolarized light	35 (10.3)
Digital dermoscopy	19 (5.6)
Digital video dermoscopy	2 (0.6)
Combination	82 (24.3)

**Table 3:** Training of dermatologists in dermatoscopy *.

**Sources of training (n = 338)**	**N (%)**
National conferences (Greece)	252 (74.5)
Residency training	203 (60)
Dermatology books	176 (52.1)
Webinars, online courses	125 (37)
International conferences	58 (17.2)
**Satisfaction for educational level (n = 338)**	**N (%)**
1 (Not sure)	0
2 (Very unsatisfied)	17 (5)
3 (Unsatisfied)	91 (27)
4 (Satisfied)	154 (45.5)
5 (Very satisfied)	76 (22.5)
**Diagnostic algorithms (n = 336)**	**N (%)**
Blink diagnosis	239 (71.1)
Dermatoscopic ABCD algorithm	201 (60)
Dermatoscopic “ugly duckling sign”	145 (43.2)
Analytic algorithms (pattern analysis)	35 (10.4)

* Questionnaire answered by 341/366 (93.2%) of dermoscopy-using dermatologists.

**Table 4:** Clinical practice of dermatoscopy *.

**Indications (diseases) (n = 338)**	**N (%)**
Dermatologic oncology	338 (100)
Nail and hair disorders	301 (89.1)
Inflammatory diseases	238 (70.4)
Cutaneous infectious conditions	191 (56.5)
**Application of dermoscopy in % of patients examined (n = 339)**	**N (%)**
<20%	32 (9.5)
20–50%	83 (24.5)
50–80%	91 (26.8)
>80%	133 (39.2)
**Application on only high-risk melanoma patients (n = 338)**	**N (%)**
Yes	11 (3.2)
No	327 (96.8)
**Avoid unnecessary surgical excisions (n = 339)**	**N (%)**
Yes	219 (64.6)
No	13 (3.8)
Several times	107 (31.6)
**Determine therapeutic approach (n = 339)**	**N (%)**
Yes	186 (54.9)
No	4 (1.2)
Several times	149 (43.9)
**Accurate biopsy guidance (n = 336)**	**N (%)**
Yes	299 (89)
No	37 (11)
**Dermatoscopy as a part of patients’ follow-up (n = 337)**	**N (%)**
Yes	325 (96.4)
No	12 (3.6)
**Help patient communication and general compliance (n = 338)**	**N (%)**
Yes	319 (94.4)
No	19 (5.6)
**Telecommunication/teleconferencing with colleagues (n = 339)**	**N (%)**
Yes	208 (61.4)
No	131 (38.6)
**Save digital images from the dermatoscopic examination (n = 338)**	**N (%)**
Yes	215 (63.61)
No	123 (36.39)
**Dermatoscopic medical history of help for patients’ follow-up (n = 337)**	**N (%)**
Yes	314 (93.18)
No	23 (6.82)
**Additional charge (n = 336)**	**N (%)**
Yes	85 (25.3)
No (part of dermatological examination)	237 (70.5)
Neither yes nor no	14 (4.2)
**Earliest diagnosis of melanoma from other specialtists (n = 337)**	**N (%)**
Yes	181(53.7)
No	156 (46.3)

* Questionnaire answered by 341/366 (93.2%) of dermoscopy-using dermatologists.

## Data Availability

Data are contained within the article and Supplementary Materials.

## Supplementary Materials

The following supporting information can be downloaded at: https://www.mdpi.com/article/10.3390/jcm13040972/s1.
