# Supplementary material for: Use of Dermoscopy among Greek Dermatologists in Everyday Clinical Practice: A National Questionnaire-Based Study
Source: J Clin Med. 2024 Feb 8;13(4):972. doi: 10.3390/jcm13040972 (PMC10888643; doi:10.3390/jcm13040972)
Supplement: Supplementary file 1 [file jcm-13-00972-s001.zip › jcm-2736385-supplementary.pdf]

Questionnaire

1.Gender

- ✓ Male
- ✓ Female

2. Age

- ✓ <30 years
- ✓ 30-50 years
- ✓ >50 years

3. Please indicate your working area.

…………………………………………………

4. In which medical facility do you currently work?

- ✓ Private practice
- ✓ General hospital
- ✓ Primary Health Care
- ✓ Both private practice and general hospital
- ✓ Other

5. Do you own a dermatoscope?

- ✓ Yes
- ✓ No, I do not care.

6. If no, fill out the reasons that prevented you.

- ✓ Financial reasons
- ✓ Lack of experience
- ✓ Lack of update and training
- ✓ Financial reasons, lack of experience
- ✓ Lack of update, training and experience
- ✓ Wrong timing
- ✓ Other subspecialty
- ✓ Different work field

If you own a dermatoscope, please continue with the next section.

7. What kind of dermatoscope do you own?

- ✓ Handheld dermatoscope with unpolarized light
- ✓ Handheld dermatoscope with polarized and unpolarized light
- ✓ Digital dermatoscopy
- ✓ Digital video-dermatoscopy
- ✓ Combination

8. How did you receive dermatoscopy training?

- ✓ National conferences (Greece)
- ✓ Residency training
- ✓ Dermatology books
- ✓ Webinars-Online Courses
- ✓ International Conferences

9. Do you believe that you have received adequate training in the use of a dermatoscopy?

- ✓ 1 (Not sure)
- ✓ 2 (Very unsatisfied)
- ✓ 3 (Unsatisfied)
- ✓ 4 (Satisfied)
- ✓ 5 (Very satisfied)

10. In what percentage of your patients do you apply dermatoscopy?

- ✓ < 20%
- ✓ 20 - 50%
- ✓ 50 - 80%
- ✓ 80%

11. What do you usually use as a diagnostic algorithm?

- ✓ Blink diagnosis
- ✓ Dermatoscopic ABCD algorithm
- ✓ Dermatoscopic "ugly duckling sign"
- ✓ Analytic algorithms (pattern analysis)

12. Does dermatoscopy reduce your average time for diagnosis?

- ✓ Yes
- ✓ No

13. Do you use dermatoscopy only in high-risk melanoma patients?

- ✓ Yes
- ✓ No

14. Specify one or more of the following indications for which you use dermatoscopy.

- ✓ Dermatologic oncology
- ✓ Nail and hair disorders
- ✓ Inflammatory diseases
- ✓ Cutaneous infectious conditions

15. Does dermatoscopy determine your treatment approach?

- ✓ Yes

- ✓ No
- ✓ Several times

16. Do you think that dermatoscopy helps you to avoid unnecessary surgical operations?

- ✓ Yes
- ✓ No
- ✓ Several times

17. Does dermatoscopy help you to obtain more accurate biopsy samples and collaborate with the pathologist?

- ✓ Yes
- ✓ No

18. Is dermatoscopy part of the follow up of your patients?

- ✓ Yes
- ✓ No

19. Do you believe that dermatoscopy and other imaging methods help patient communication and general compliance?

- ✓ Yes
- ✓ No

20. Do you save your digital images from the dermatoscopic examination?

- ✓ Yes
- ✓ No

21. Do you think the dermatoscopic medical history would be helpful?

- ✓ Yes
- ✓ No

22. Do you use dermatoscopy as a mean for telecommunication/teleconferencing with your colleagues to share diagnostic approaches?

- ✓ Yes
- ✓ No

23. Do you believe that the training of other specialties (general practitioners, plastic surgeons, pediatricians, physicians) would contribute to the earliest diagnosis of melanoma?

- ✓ Yes
- ✓ No

24. Do you think the dermatoscopy examination should be charged separately?

- ✓ Yes

- ✓ No,
- ✓ Other
